# Transketolase-mediated erythrose-4-phosphate provides an essential source for anthocyanin biosynthesis in petunia

**DOI:** 10.1093/hr/uhaf285

**Published:** 2025-12-22

**Authors:** Xin Li, Wenjie Yang, Jiahao Cao, Wenqi Deng, Chenxi Wang, Yi Yao, Weiyuan Yang, Yixun Yu, Shiwei Zhong, Juanxu Liu

**Affiliations:** College of Horticulture, South China Agricultural University, Guangzhou 510642, China; College of Horticulture, South China Agricultural University, Guangzhou 510642, China; College of Horticulture, South China Agricultural University, Guangzhou 510642, China; College of Horticulture, South China Agricultural University, Guangzhou 510642, China; College of Horticulture, South China Agricultural University, Guangzhou 510642, China; College of Horticulture, South China Agricultural University, Guangzhou 510642, China; Chinese Academy of Science, Key Lab Plant Resources Conservation & Sustainable Utilization, South China Botanical Garden, Guangzhou 510650, China; College of Horticulture, South China Agricultural University, Guangzhou 510642, China; College of Horticulture, South China Agricultural University, Guangzhou 510642, China; College of Horticulture, South China Agricultural University, Guangzhou 510642, China

## Abstract

The shikimate pathway is critical for the biosynthesis of aromatic amino acids and a diverse array of secondary metabolites in plants, including anthocyanins. Erythrose-4-phosphate (E4P) serves as a crucial precursor in the shikimate pathway. Transaldolase (TA) and transketolase (TK) are two pivotal enzymes involved in E4P synthesis in plants through the oxidative pentose phosphate pathway (OPPP) and Calvin cycle pathways. During the coloring stage of flowers, a large number of anthocyanins accumulate. However, the source of E4P required for anthocyanin accumulation is still unknown. In this study, we characterized the TA and TK family members in petunia (*Petunia hybrida*), an important ornamental plant. Virus-induced gene silencing (VIGS) and RNAi techniques indicated that *PhTA1* or *PhTA2* silencing did not lead to visible phenotype change in petunia, while cosilencing of *PhTK1-TK2* resulted in significantly lighter colors in flowers and leaves. The levels of anthocyanins, chlorophyll, E4P, flavonoids, and three aromatic amino acids all significantly decreased in *PhTK1-TK2*-silenced plants compared with the control. Additionally, cosilencing of *PhTK1 and PhTK2* disrupted the flavonoid metabolome profile in petunia flowers. In summary, PhTK1 and PhTK2 provide the primary E4P source for anthocyanin biosynthesis.

## Introduction

In plants, the aromatic amino acids (AAA), phenylalanine (Phe), tyrosine (Tyr), and tryptophan (Trp), play central roles in metabolic processes and are synthesized from the shared precursor metabolite chorismate, which is derived from the shikimate pathway [1]. Erythrose-4-phosphate (E4P) and phosphoenolpyruvate (PEP) serve as the precursors for the synthesis of the shikimate pathway in plants [2–4]. The shikimate pathway involves seven steps. Initially, PEP and E4P are utilized to generate 3-deoxy-d-arabino-heptulosonate-7-phosphate (DAHP) under the catalysis of DAHP synthase (DAHPS). The next six steps require the catalysis of five enzymes to form chorismate, including 3-dehydroquinate synthase (DHQS), 3-dehydroquinate dehydratase (DHQ/SDH), shikimate kinase (SK), 5-enolpyruvylshikimate-3-phosphate synthase (EPSPS), and chorismate synthase (CS).

E4P is generated primarily through two pathways [2, 3] (Fig. S1). E4P can be synthesized by transketolase (TK) as part of the Calvin cycle or by either transaldolase (TA) or TK through the oxidative pentose phosphate pathway (OPPP). In the OPPP, E4P can be synthesized under the catalytic activity of TA using sedoheptulose 7-phosphate (S7P) and glyceraldehyde 3-phosphate (GAP) as substrates. Additionally, TK can synthesize E4P and xylulose 5-phosphate (Xu5P) from fructose 6-phosphate (F6P) and GAP. Moreover, TK is additionally required in the Calvin cycle, in which it converts GAP and F6P to Xu5P and E4P [5, 6].

TA is widely present in archaea, microorganisms, plants, and animals [7–10]. Two TA members localized in the chloroplasts have been identified in *Arabidopsis thaliana* and *Solanum lycopersicum* [10, 11]. Tomato ToTal1 and ToTal2 show a distant evolutionary relationship. *ToTal1* is primarily expressed in the roots, stems, and mature fruits, while *ToTal2* has a higher expression level in the stems and cotyledons, and a lower expression level in mature leaves and flowers [10]. The *Arabidopsis gsm2* mutant (a *ToTal1* homolog) displays glucose hypersensitivity leading to leaf yellowing and reduced thylakoids, despite normal growth under nonstress conditions, with the phenotype being rescued by phenylalanine [12]*.* Unlike TA, TK can catalyze the formation of E4P not only through the OPPP but also via the Calvin cycle. The substrates and products involved in reactions catalyzed by TK serve as precursors for phenylpropanoid metabolism (E4P), nucleotide synthesis (pentose phosphate), carbohydrate synthesis (F6P), and the lower part of glycolysis (GAP) [13]. These compounds further participate in respiratory metabolism, as well as amino acid and lipid synthesis [2, 14]. Research on TK has been reported in yeast [15], bacteria [16], animals [17], and humans [18]. However, studies on TK in plants are still relatively limited. In tobacco, it was found that inhibiting TK activity significantly reduced photosynthetic efficiency, with only the chlorophyll content in the midrib of the leaves declining [19]. In cucumbers, the antisense expression of *TK* led to slow growth, reduced proportion of female flowers, and thus decreased yield, while also inhibiting transpiration rates and photosynthesis [20].

Phe is the key precursor of anthocyanin biosynthesis, which plays an important role in the formation of flower color, plant stress tolerance, attraction of animals, and pollinating insects [21]. Many ornamental plants synthesize large amounts of anthocyanins during the flower coloring stage [22]. Our previous studies have shown that the key genes of the shikimate pathway (*PhENO1*, *PhPPTs*, *PhCS*, and *PhSK*) in petunia (*Petunia hybrida*) are involved in anthocyanin synthesis [23–25]. E4P is a precursor of the shikimate pathway. However, it is still unknown whether the synthesis of E4P affects anthocyanin content and the source of E4P required for anthocyanin synthesis. In this study, we utilized petunia, a model plant commonly used in research on flower color formation, as the experimental material to investigate the source of E4P in the anthocyanin biosynthetic pathway. The results from RNA interference (RNAi) and virus-induced gene silencing (VIGS) indicated that *PhTA1* or *PhTA2* silencing did not result in any visible phenotypic changes in petunia. In contrast, *PhTK1-TK2* silencing led to significant yellowing of the leaves, and the flower color changed from deep purple to light pink. The contents of E4P, shikimate, Phe, total flavonoids, anthocyanins, chlorophyll levels, and photosynthetic efficiency were all significantly reduced in the *PhTK1-TK2*-silenced plants compared with the control. Metabolomic analysis of petunia corollas revealed that *PhTK1-TK2* silencing changes the flavonoid metabolome profile.

## Results

### Identification of *PhTA and PhTK* gene families in petunia

**Figure 1:**
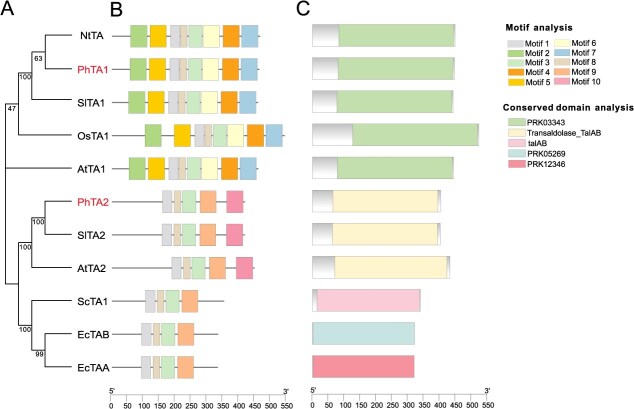
Phylogenetic tree (A), motif (B), and protein conserved domains (C) analyses of TA members among different species. Accession numbers in the GenBank/DDBJ databases are as follows. *Nicotiana tabacum* NtTA (NP_001312459.1), *P. hybrida* PhTA1 (Peaxi162Scf00842g00319.1) and PhTA2 (Peaxi162Scf00002g00815.1), *S. lycopersicum* SlTA1 (AF184164.1), *Oryza sativa* OsTA1 (BAB92890.1), *A. thaliana* AtTA1 (AT5G13420.1) and AtTA2 (AT1G12230.2), *S. lycopersicum* SlTA2 (AY007225.1), *Saccharomyces cerevisiae* ScTA1 (P15019.4), and *Escherichia coli* EcTAA (NP_289016.1) and EcTAB (NP_414549.1).

To identify the homologous genes of TA in petunia, a basic local alignment search tool (BLAST) search was performed using *Arabidopsis* TA as the query. The coding sequence (CDS) lengths of *PhTA1* and *PhTA2* were 1197 and 1326 bp, respectively. PhTA1 was predicted to be localized in the chloroplasts, and PhTA2 was predicted to be localized in the chloroplasts and mitochondria (Cell-PLoc 2.0). The homologous similarity between PhTA1 and PhTA2 is relatively low, at only 27.00%, suggesting that PhTAs may undergo functional differentiation in petunia. Phylogenetic analysis indicated that the TA family was primarily divided into two subfamilies. PhTA1 clustered with NtTA1, OsTA1, and SlTA1 in the TA1 subfamily, while PhTA2 grouped with AtTA2 and SlTA2 in the TA2 subfamily (Fig. 1A). Analysis of the motifs and conserved domains within the TA family revealed that all members of the TA1 subfamily contain eight motifs, with motifs 2, 5, and 7 appearing exclusively in the TA1 subfamily and motif 10 appearing only in the TA2 subfamily (Fig. 1B). The conserved protein domain of proteins within different subfamilies varies considerably. The TA1 subfamily exhibited greater conservation, with all members containing a ‘PRK03343’ domain. In contrast, the TA2 subfamily showed lower conservation, with different species harboring four distinct conserved domains (Fig. 1C). Analysis of the cis-acting elements of their promoters showed that *PhTA1* and *PhTA2* contained 18 cis-acting elements, more than half of which were related to light response (Fig. S2A).

**Figure 2:**
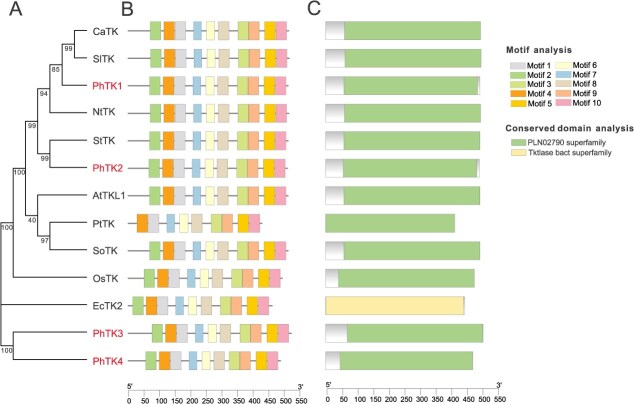
Phylogenetic tree (A), motif (B), and protein conserved domains (C) analyses of TK members among different species. Accession numbers in the GenBank/ DDBJ databases are as follows. *Capsicum annuum* CaTK (XP_016572652.1), *S. lycopersicum* SlTK(XP_004240048.2), *P. hybrida* PhTK1 (Peaxi162Scf00959g00213), PhTK2 (Peaxi162Scf00238g00810), PhTK3 (Peaxi162Scf01227g00034), and PhTK4 (Peaxi162Scf01227g00027), *N. tabacum* NtTK (NP_001312893.1), *Solanum tuberosum* StTK (NP_001275202.1), *A. thaliana* AtTKL1 (NP_567103.1), *Persicaria tinctoria* PtTK (BAB62078.1), *Spinacia oleracea* SoTK (O20250.1), *O. sativa* OsTK(CAH66254.1), and *E. coli* EcTK2 (HDP9345749.1).

Similarly, using the BLAST method, we identified four members of the petunia *PhTKs* family, *PhTK1* (Peaxi162Scf00959g00213), *PhTK2* (Peaxi162Scf00238g00810), *PhTK3* (Peaxi162Scf01227g00034), and *PhTK4* (Peaxi162Scf01227g00027). The CDS length of all four members exceeded 2000 bp, with *PhTK3* having the longest CDS at 2271 bp and *PhTK4* having the shortest at 2121 bp. Phylogenetic analysis revealed that PhTK1 and PhTK2 are closely related and cluster with NtTK, whereas PhTK3 and PhTK4 form a separate branch that is distantly related to TK members from other species (Fig. 2A). Based on our previous RNA-seq data [26], we found that the expression level of both *PhTK3* and *PhTK4* was much lower than that of *PhTK1* and *PhTK2* in both flowers and leaves (Table S1).

The sequence similarity between PhTK1 and PhTK2 was as high as 88.14%. PhTK1 only shares 48.88% and 47.65% amino acid sequence identity with PhTK3 and PhTK4, respectively, and PhTK3 shares 81.05% amino acid sequence identity with PhTK4. An analysis of the motifs and conserved protein domains in TK members showed that the number of motifs ranged from 9 to 10, and all contained a ‘PLN02790 superfamily’ conserved domain in plants (Fig. 2B and C). Analysis of the cis-acting elements of the four *PhTKs* promoters indicated that all four promoters contained a significant number of light-responsive elements, in addition to elements related to hormones and stress responses (Fig. S2B).

### Subcellular localization of PhTAs and PhTKs

**Figure 3:**
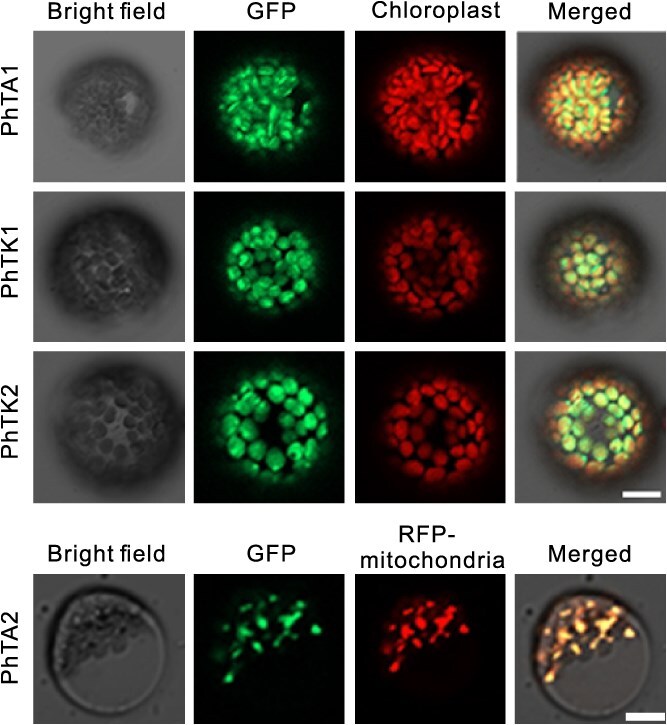
Subcellular localization of PhTAs, PhTK1 and PhTK2. The scale bar = 10 μm.

To investigate the function of the *TA* and *TK* gene families, we examined the subcellular localization of their respective proteins. The vectors were generated by fusing the CDS of each gene with green fluorescent protein (GFP). Due to the very low expression levels of *PhTK3* and *PhTK4*, and their distant phylogenetic relationship with the known functional NtTK, we focused specifically on PhTK1 and PhTK2. The constructed vectors were introduced into the petunia protoplasts. After 24 h, the fluorescence signals were detected by using laser confocal microscopy. The results showed that, except for PhTA2-GFP, the green fluorescence signals emitted by PhTA1-GFP, PhTK1-GFP, and PhTK2-GFP overlapped with the fluorescence signals emitted by chloroplasts, indicating that PhTA1, PhTK1, and PhTK2 are localized in the plastid (Fig. 3). The PhTA2-GFP fluorescence signal exhibited punctate distribution. The green fluorescence signal of PhTA2-GFP colocalized with the red fluorescence emitted by the mitochondrial marker (Fig. 3). These results indicated that PhTA2 is localized in the mitochondria.

### The expression pattern of *PhTAs, PhTK1*, and *PhTK2*

**Figure 4:**
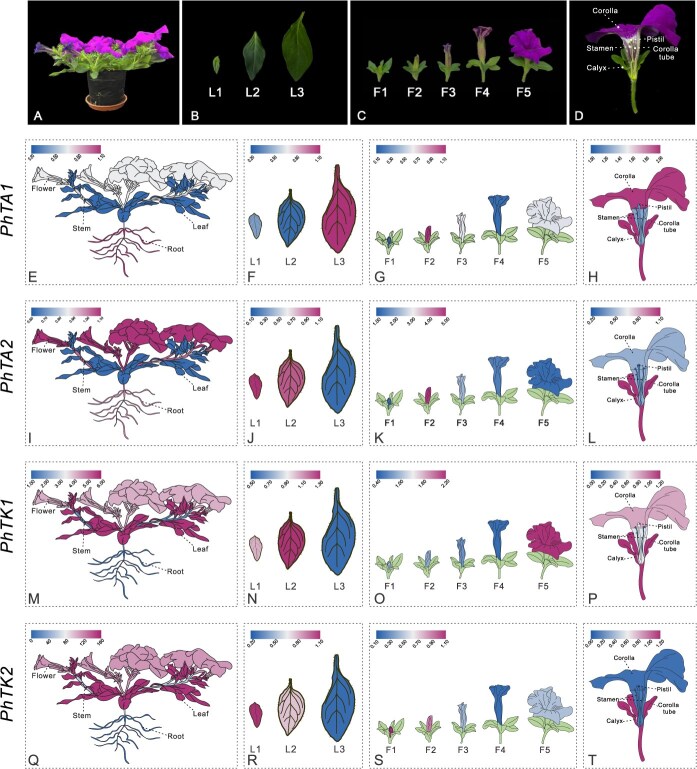
A series of fancy heatmaps exhibiting the expression patterns of *PhTA1*, *PhTA2*, *PhTK1*, and *PhTK2* determined by qPCR. (A) Photos of petunia growing in pots. (B) Three stages of leaf development. (C) Five stages of flower development. (D) Structure of the petunia flower. Expression of *PhTA1*(E), *PhTA1*(I), *PhTK1* (M), and *PhTK2* (Q) in different organs (root, stem, leaf, and flower). Leaf development was divided into three stages: L1 (length 2.5 cm), L2 (4.0 cm), and L3 (7.0 cm). Expression of *PhTA1*(F), *PhTA1*(J), *PhTK1*(N), and *PhTK2* (R) in different leaf development stages. Flower development was divided into five stages: F1 (length 0.5 cm), F2 (1.0 cm), F3 (2.0 cm, coloring stage of the flower), F4 (3.0 cm), and F5 (flowering stage). Expression of *PhTA1*(G), *PhTA2*(K), *PhTK1*(O), and *PhTK2* (S) in different flower development stages. Expression of *PhTA1*(H), *PhTA2*(L), *PhTK1*(P), and *PhTK2* (T) in different flower parts (corolla, corolla tube, stamen, pistil, and calyx). The legend shows the relative expression level.

Employing *PhCYP* as an internal reference gene, we evaluated the expression levels of *PhTA1*, *PhTA2*, *PhTK1*, and *PhTK2* in various organs (roots, stems, leaves, and flowers) across five flower development stages and five flower tissues of petunia. Distinct expression patterns were observed between the *TA* and *TK* families across different organs. Both *PhTA1* and *PhTA2* exhibited higher expression levels in the roots and lowest in the leaves (Fig. 4E and I; Fig. S3A and E). In contrast, *PhTK1* and *PhTK2* showed similar expression patterns. Both of them showed the lowest expression levels in the roots and the highest in the leaves (Fig. 4M and Q; Fig. S3I and M). Among the three stages of leaf development, except for higher expression of *PhTA1* at stage L3, *PhTA2*, *PhTK1*, and *PhTK2* all exhibited higher expression levels in the early stages of leaf development (Fig. 4F, J, N, and  R; Fig. S3B, F, J, and  N). During the five stages of flower development, both members of the *PhTA* family show the highest expression levels at stage F2, *PhTK1* peaked at stage F5, while *PhTK2* peaked at F1 (Fig. 4G, K, O, and  S; Fig. S3C, G, K, and  O). In the five different parts of the flower, the *PhTA* and *PhTK* families demonstrated similar expression trends. All four genes showed the highest expression levels in the sepals, with *PhTA1* and *PhTK1* also showing relatively high expression in the corollas, while *PhTA2* and *PhTK2* exhibited lower expression levels in the corollas (Fig. 4H, L, P, and  T; Fig. S3D, H, L, and  P).

### VIGS- and RNAi-mediated *PhTK1-TK2* silencing changed leaf and flower color

We have previously established an efficient and convenient virus-induced gene silencing (VIGS) system [27, 28]. Therefore, we utilized VIGS technology to conduct preliminary studies on the functions of *PhTA1*, *PhTA2*, *PhTK1*, and *PhTK2*. Using the cDNA of the petunia ‘Ultra’ as a template, we selected approximately 300-bp specific sequences of *PhTA1*, *PhTA2*, *PhTK1*, and *PhTK2* and cloned them into the pTRV2 vector. The previously constructed pTRV2-GFP vector was used as a control vector [27, 28]. Thirty to 35 petunia plants were infected with each vector.

**Figure 5:**
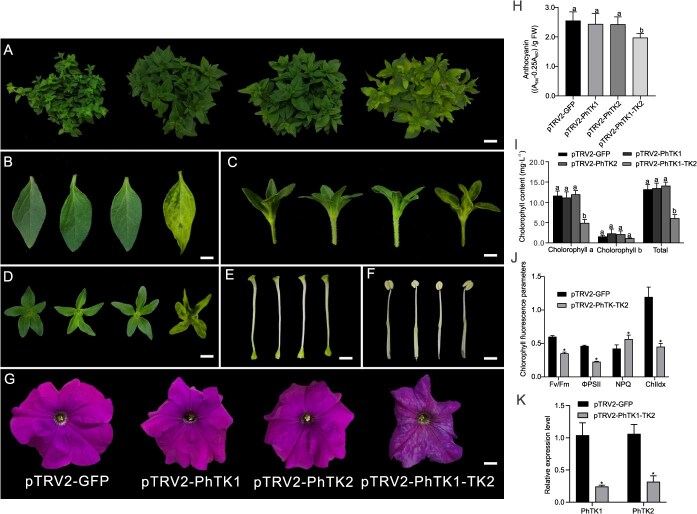
The phenotype of VIGS-mediated silencing of *PhTK1*, *PhTK2*, and *PhTK1-TK2.* Left to right: pTRV2-GFP (control), *PhTK1*-, *PhTK2*-, and *PhTK1-TK2*-silenced plants: top view (A), leaves (B), front view of calyx (C), top view of calyx (D), pistils (E), stamens (F), and flowers (G). Scale bars = 3 cm in (A), 2 cm in (B), 1 cm in (C) and (D), 0.5 cm in (E) and (F), and 2 cm in (G). Anthocyanin (H) and chlorophyll (I) content in *PhTK1*-, *PhTK2*-, and *PhTK1-TK2*-silenced plants. (J) Changes in photosynthetic efficiency indexes of leaf blades in *PhTK1*-*TK2*-silenced plants. Fv/Fm, maximum quantum yield of PSII. ΦPSII, photosynthetic quantum yield of photosystem II. NPQ, nonphotochemical quenching. ChlIdx, chlorophyll index. Data in the graphs are expressed as mean ± SD (*n* = 9). Different lowercase letters and ‘*’ indicate significant differences at the *P* = 0.05 level.

The phenotypes of the petunia plants were observed about 1 month later. Surprisingly, we found that individual silencing of *PhTA1*, *PhTA2*, *PhTK1*, or *PhTK2* did not lead to any visible phenotype change of petunia (Fig. 5; Fig. S4). Given the different subcellular localization of PhTA1 and PhTA2, as well as the common subcellular localization of PhTK1 and PhTK2 in the plastid, it cannot be ruled out that *PhTK1* and *PhTK2* exhibit functional redundancy. We constructed the pTRV2-PhTK1-TK2 vector to cosilence *PhTK1* and *PhTK2.* The VIGS results showed that *PhTK1 and PhTK2* cosilencing (*PhTK1-TK2* silencing) resulted in significant yellowing of the leaves and calyxes, and the corolla color lightened considerably, exhibiting a water-soaked appearance (Fig. 5). The total chlorophyll content in the yellowing leaves decreased significantly by 53.71% compared with the control (Fig. 5I), and the anthocyanin content in the corollas significantly reduced by 15.96% (Fig. 5H). In addition, we found that *PhTK1*-*TK2* silencing significantly reduced the photosynthesis-related parameters Fv/Fm, ΦPSII, and ChlIdx by 42.79%, 51.22%, and 62.08%, respectively, while the NPQ increased significantly by 1.31 times compared with the control (Fig. 5J). The results of quantitative real-time PCR (qPCR) analysis showed that the expression level of *PhTK1* and *PhTK2* in the pTRV2-PhTK1-TK2 treated plants decreased significantly by 75.73% and 70.25%, respectively, compared with the control, confirming VIGS-mediated cosilencing of *PhTK1* and *PhTK2* (Fig. 5K). Furthermore, we silenced *PhTK3* and *PhTK4* to investigate whether the low expression of *PhTK3* and *PhTK4* also affected the flower and leaf color. Due to their high homology, we chose a homologous region of both genes for silencing. The results showed that silencing *PhTK3-TK4* did not lead to a visible phenotype (Fig. S5). Therefore, we mainly focused on the function of *PhTK1* and *PhTK2* in our subsequent research.

**Figure 6:**
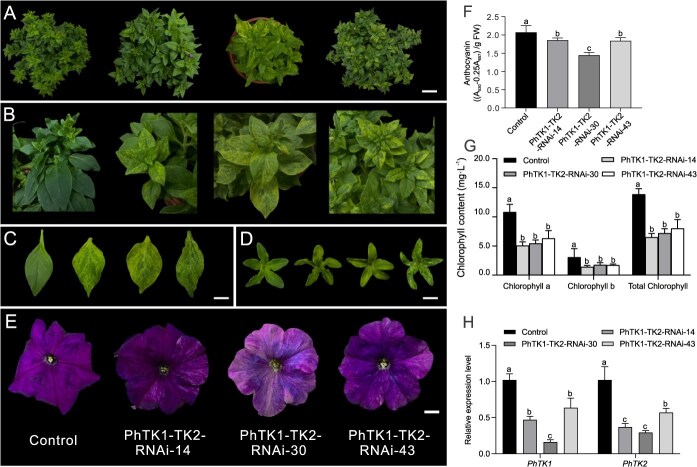
Phenotypic alterations caused by RNAi-mediated silencing of *PhTK1-TK2.* Left to right: control, *PhTK1-TK2*-RNAi-14, *PhTK1-TK2*-RNAi-30, and *PhTK1-TK2*-RNAi-43 lines: top view (A), partial enlargement (B), leaves (C), top view of calyx (D), and flowers (E). Scale bars = 4 cm in (A), 2 cm in (C) and (D), and 1 cm in (E). Anthocyanin (F) and chlorophyll (G) content in *PhTK1-TK2*-RNAi plants. (H) The expression levels of *PhTK1* and *PhTK2* in *PhTK1-TK2*-RNAi plants. Data in the graphs are expressed as mean ± SD (*n* = 9). ‘*’ indicates significant differences at the *P* = 0.05 level.

To reinforce the experimental results of VIGS, we generated *PhTK1-TK2*-silenced transgenic petunia plants by RNAi technology. Approximately 3 months after infection, a total of 83 resistant seedlings were obtained; among them, PCR analysis showed that 34 seedlings were positive plants (Fig. S6). The 34 seedlings were transplanted from the culture bottles into pots, and 4 weeks later, 13 plants exhibited phenotypes consistent with *PhTK1*-*TK2*-silenced plants. The leaves and calyxes turned significantly yellow, and the flower color became noticeably lighter (Fig. 6). Analysis of chlorophyll content in the *PhTK1*-*TK2*-RNAi-14, -30, and -43 lines indicated that chlorophyll levels decreased significantly by 53.15%, 48.11%, and 42.22%, respectively, compared with the control (Fig. 6G). The anthocyanin content was also significantly reduced by 10.51%, 30.41%, and 11.31% compared with the control, respectively (Fig. 6F). qPCR analysis showed that the expression level of *PhTK1* and *PhTK2* in the three *PhTK1*-*TK2*-RNAi lines was significantly decreased compared with the control (Fig. 6H).

### *PhTK1-TK2* silencing led to a decrease of E4P, three aromatic amino acids, and flavonoids in petunia

We further determined various downstream metabolites of TK in the *PhTK1-TK2*-RNAi-30 plants. Initially, we measured the content of E4P, the direct product of TK, and observed a significant decrease in the *PhTK1-TK2*-silenced plants compared with the control (Fig. S7A). We further examined the amino acid content in *PhTK1*-*TK2*-silenced plants. The results showed that *PhTK1*-*TK2* silencing resulted in a significant decrease in the levels of Phe, Tyr, and Trp, by 87.10%, 84.59%, and 93.19%, respectively, while the total amino acids were increased in *PhTK1-TK2*-silenced plants (Fig. S7B). Moreover, cosilencing of *PhTK1* and *PhTK2* led to a notable 26.06% reduction in flavonoid levels compared with the control (Fig. S7C).

### *PhTK1-TK2* silencing disturbed the flavonoid metabolome profile

**Figure 7:**
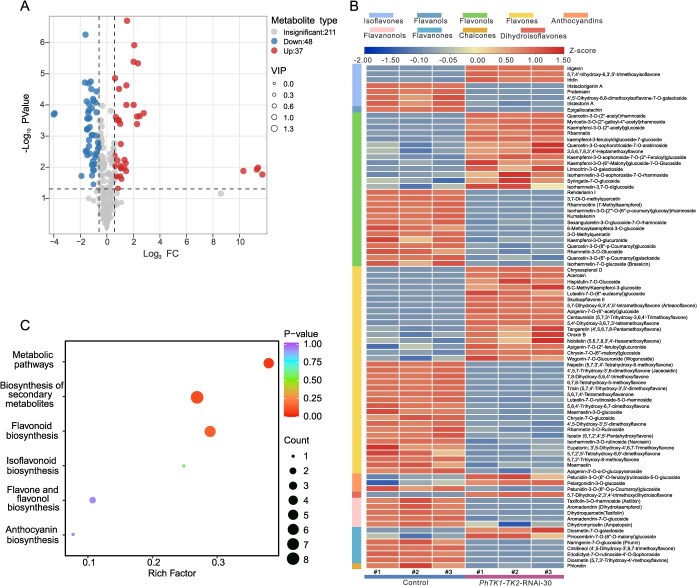
Analysis of differential metabolites in the corollas of *PhTK1-TK2*-silenced plants compared with controls. (A) Volcano plot of detected metabolites. (B) Heat map of differential metabolites. The different colored squares on the left represent different kinds of flavonoids. (C) KEGG enrichment analysis.

Through colorimetric analysis, we observed that *PhTK1-TK2* silencing leads to a significant decrease in anthocyanins (Figs 5H and 6F). Furthermore, we conducted metabolomics analysis of flavonoids between the corollas of *PhTK1-TK2*-RNAi-30 and control plants, which provided absolute quantification of individual anthocyanin metabolites. Differential metabolites were screened and kept when the fold change value was ≥1.5 or ≤0.67 and the VIP value was ≥1. A total of 85 differential metabolites were identified among 296 flavonoid metabolites, with nine different types of flavonoid metabolites (Table S6). Among these, 48 metabolites were significantly downregulated and 37 were significantly upregulated in the *PhTK1*-*TK2*-silenced group (Fig. 7A and B). The most downregulated metabolites in the *PhTK1*-*TK2*-silenced plants were observed in the flavone group, particularly 5,7,2′-trihydroxy-8-methoxyflavone (~94%), while the most upregulated was found in the flavonol group, particularly 6-*C*-methylkaempferol-3-glucoside (~12-fold). Among the nine different types of flavonoid metabolites, flavones and flavonols had the most differential metabolites, with 35 and 26, respectively. In addition, we identified three differentially expressed anthocyanins, among which petunidin-3-*O*-(6″-*O*-feruloyl)rutinoside-5-*O*-glucoside and pelargonidin-3-*O*-glucoside showed significant increases of 2.75-fold and 2.16-fold, respectively, in the *PhTK1-TK2*-silenced group, while petunidin-3-*O*-(6″-*O*-p-coumaroyl)glucoside decreased by 64.00% compared with the control. KEGG enrichment analysis revealed that the above differential metabolites were mainly enriched in ‘Biosynthesis of secondary’, ‘Metabolic pathways’, and ‘Flavonoid biosynthesis’ (Fig. 7C). In conclusion, *PhTK1*-*TK2* silencing changed the flavonoid metabolome profile in petunia flowers.

### The silencing of the other genes involved in the Calvin cycle did not change flower color

Since *PhTA1* silencing did not result in significant phenotypic changes, we hypothesize that *PhTK1* and *PhTK2* provide the necessary E4P source for anthocyanin formation through the Calvin cycle rather than the OPPP. To exclude the possibility that the decrease in anthocyanin levels caused by *PhTK1-TK2* silencing was not due to a reduction of other metabolites, we silenced five genes encoding the enzymes upstream and downstream of TK in the Calvin cycle, *PhRPI* (Peaxi162Scf00274g00342), *PhRPE* (Peaxi162Scf00282g00110), *PhSBPase* (Peaxi162Scf00957g00328), *PhFBA* (Peaxi162Scf00083g01223), and *PhTPI* (Peaxi162Scf00282g00110). The results showed that silencing the five key genes of the Calvin cycle did not lead to changes in flower color in petunias; instead, we observed a significant lightening of leaf color, which is consistent with the phenotypic changes in leaf color resulting from PhTK1-TK2 silencing (Figs S8 and S9).

## Discussion

The shikimate pathway is a key upstream pathway of the anthocyanin pathway, providing essential precursor sources for anthocyanin synthesis. In this study, we found that the synthesis of E4P affects the content of anthocyanins in petunia flowers, and we found that the main source of E4P for anthocyanin synthesis was catalyzed by TK. Additionally, TK was also involved in the leaf color formation in petunia.

*Arabidopsis gsm2* mutant exhibits severe glucose hypersensitivity phenotypes, including chlorotic cotyledons and stunted growth that worsen with increasing glucose levels [12]. Yang *et al.* reported that rice plants with a *TA* knockout displayed a dwarfed phenotype with altered leaf morphology and culm elongation [9]. Another TA mutant, *tra2*, exhibited low lignin content and increased saccharification efficiency, indicating its role in diverting carbohydrate flux toward phenolic metabolism [11]. In this study, plastid-localized *PhTA1* or mitochondria-localized *PhTA2* silencing did not affect the growth and development of petunias. *PhTA1* was most highly expressed in the roots, with lower expression levels in the surface organs, suggesting that *PhTA1* could play a limited role in the developmental processes of flowers and leaves. *PhTA2* exhibited high expression levels in flowers and stems, and the shikimate pathway occurs in plastids, whereas *PhTA2* is localized in mitochondria, suggesting that PhTA2 is not involved in the shikimate pathway. Next, we will identify the function of *PhTA2* in mitochondria using gene editing technology.

In our study, we found that individual silencing of *PhTK1* or *PhTK2* did not show a significant change in petunia, while cosilencing *PhTK1* and *PhTK2* led to lighter flower color. These results indicated that *PhTK1* and *PhTK2* are involved in the formation of flower color and exhibit functional redundancy. These results were consistent with our previous findings that inhibition of key enzymes or reduction of compounds in the shikimate pathway can lead to a decrease in downstream anthocyanins [23–25]. Our study also showed significant reductions in the contents of E4P, three aromatic amino acids, flavonoids, and anthocyanins, as well as a change in the profile of flavonoid metabolome. The metabolomics analysis provided absolute quantification of individual compounds. Although total anthocyanin content decreased in *PhTK1*-*TK2*-silenced plants, we observed a significant increase in certain individual anthocyanins, such as pelargonidin-3-*O*-glucoside. This suggests that the loss of TK function not only reduces flux into the phenylpropanoid pathway but also triggers a reprogramming of flavonoid metabolism. Moreover, our previous studies have also revealed that partial reprogramming of flavonoid metabolism also occurred in silencing the key genes in the anthocyanin pathway (*Ph5GT*) or the shikimate pathway (*PhSK*) [25, 29]. In this study, standard thresholds (FC ≥1.5 or ≤0.67 and VIP ≥1) were used to identify metabolites with differential accumulation, prioritizing statistical stringency and large fold changes. While this approach effectively highlights the most pronounced metabolic alterations, it may inadvertently exclude metabolites with more modest yet biologically significant changes in abundance. Future targeted analyses could be designed to explore these subtler fluctuations and provide an even more comprehensive view of the metabolic network influenced by *PhTK*, and in Table S6, the content of all metabolites in the control and *PhTK1-TK2*-silenced corollas was provided. Since the silencing of *PhTA1* did not result in significant phenotypic changes, we hypothesize that TK-mediated Calvin cycle flux provides the primary E4P source for anthocyanin biosynthesis. Silencing other genes in the Calvin cycle did not affect flower color, implicating E4P as the metabolite responsible for the color change. This implies that TK could serve as the pivotal enzyme in this pathway, and there may be potential isoenzymes or alternative pathways upstream of TK. Based on these results, we infer that the lightening of flower color following *PhTK1-TK2* silencing was due to the lack of E4P rather than a nutritional deficiency caused by the lightening of leaf color.

Previous studies on TK mainly focus on its regulation of photosynthetic efficiency in the Calvin cycle. In *Craterostigma plantagineum*, there was a local loss of chlorophyll and carotenoids on the midrib when TK activity was inhibited [30]. In tobacco, inhibition of TK activity led to slight chlorosis on the midrib of leaves [19]. Previous studies have also shown that overexpressing *TK* in tobacco leads to chlorosis in leaves and significantly inhibits overall plant development [31]. However, profiling of phenylpropanoids and amino acids in *TK*-overexpressed plants showed no significant alterations in either intermediate metabolites or final products derived from the shikimate pathway [31]. In our study, the phenotype of yellowing leaves upon silencing PhTK1-TK2 is similar to that of tobacco or *C. plantagineum*. *PhTK1-TK2* silencing did not lead to growth inhibition in petunia, unlike what was observed in tobacco [19]. The above results show that the role of TK in chlorophyll metabolism or leaf color formation is highly conserved, whereas its involvement in secondary metabolic pathways, such as amino acid metabolism, exhibits subtle species-specific variations.

In summary, TK, rather than TA, catalysis provides an important source of E4P required for anthocyanin synthesis. Cosilencing of *PhTK1* and *PhTK2* simultaneously modulates both flower and leaf coloration, which may have high value in breeding for improving the color and leaf color of ornamental plants. This discovery provides a compelling strategy for creating novel cultivars with coordinated aesthetic traits, for example, plants with lighter pink flowers complemented by attractive golden-yellow foliage, all through the manipulation of one key metabolic gateway.

## Materials and methods

### Plant materials

*Petunia hybrida* ‘Ultra’ plants were grown in a greenhouse under controlled conditions (23°C ± 2°C, with 14-h light/10-h dark photoperiod). During the vegetative stage, when plants reached approximately 25 cm in height, root, stem, and leaf tissues were collected. Flowers were sampled at full bloom (corolla reflexed to 90°). All samples were rapidly flash frozen in liquid nitrogen and stored at −80°C for subsequent analyses.

### RNA extraction, RT–PCR, and *PhTAs* and *PhTKs* gene cloning

Total RNA was isolated from petunia tissues following the protocol described by Liu *et al.* [32]. Reverse transcription of mRNA was carried out using a commercially available kit (R312; Vazyme, Nanjing, China). The full-length cDNA sequences of *PhTA1* (Peaxi162Scf00842g00319), *PhTA2* (Peaxi162Scf00002g00815), *PhTK1* (Peaxi162Scf00959g00213), and *PhTK2* (Peaxi162Scf00238g00810) were cloned from *P. hybrida* ‘Ultra’ using gene-specific primers (Table S1) designed based on the *Petunia axillaris* genome available from the Sol Genomics Network (SGN) database.

### Sequence analysis

Multiple sequence alignments were performed, and a phylogenetic tree was constructed using DNAMAN and MEGA 10.1.2. Nucleotide and deduced amino acid sequence identities were evaluated using the NCBI BLAST web service and TBtools [33, 34]. InterPro software [35] was used to predict the protein conserved domain. To gain a more comprehensive understanding of PhTAs and PhTKs’ functions, conserved motifs were identified using the MEME suite. The parameters included a setting of 0 to 1 for the number of repetitions and a maximum of 10 motifs. The genomic sequences and annotation information were retrieved from the Sol Genomics Network database (https://solgenomics.net/). The promoter regions, defined as 2000-bp upstream of the transcription start sites (TSS) of *PhTAs* and *PhTKs*, were extracted for subsequent analysis. Putative cis-acting regulatory elements within these promoters were predicted using the online tool PlantCARE.

### Subcellular localization

The method for subcellular localization was conducted as previously described [27–29]. Full-length cDNA sequences of *PhTA1*, *PhTA2*, *PhTK1*, and *PhTK2* were amplified by PCR and cloned into the pSAT-1403TZ vector, which harbors GFP fusion constructs driven by the CaMV35S promoter. The primer sequences utilized for subcellular localization analysis are listed in Table S2. Constructs were verified by sequencing to confirm in-frame coding sequences and the absence of mutations. Protoplasts were isolated from petunia leaves, and polyethylene glycol-mediated transfection was conducted as previously described. Fluorescence was observed after 24-h dark incubation, using a Zeiss LSM800 confocal microscope. The excitation/emission wavelengths for GFP and RFP were 488/535 and 488/637 nm, respectively.

### Quantitative real-time PCR assays

qPCR assays were performed following established protocols [32], and the analyses complied with the MIQE guidelines for qPCR data publication [36]. *Cyclophilin* (*CYP*) (accession no. EST883944) served as the internal reference gene for quantifying cDNA abundance [37]. The results are presented as relative expression values calculated using the 2^−ΔΔCt^ method. Primer sequences utilized for qPCR analysis are detailed in Table S3. Each treatment included three biological replicates, with each replicate containing three technical repeats.

### Agroinoculation of pTRV vectors

The pTRV2-PhTA1, pTRV2-PhTA2, pTRV2-PhTK1, pTRV2-PhTK2, pTRV2-PhTK1-TK2, pTRV2-PhTK3-TK4, pTRV2-PhRPI, pTRV2-PhRPE, pTRV2-PhSBPase, pTRV2-PhFBA, and pTRV2-PhTPI vectors were constructed by amplifying approximately 300 bp of the 3′ untranslated region of *PhTA1*, *PhTA2*, *PhTK1*, *PhTK2*, and *PhTK1-TK2* (the conserved regions of *PhTK1* and *PhTK2*), and *PhRPI*, *PhRPE*, *PhSBPase*, *PhFBA*, and *PhTPI* using specific primers listed in Table S4 and inserting them into the pTRV2 vector. pTRV1 and pTRV2 vectors were transformed into *Agrobacterium tumefaciens* (strain GV3101) according to the protocol [38]. Virus-induced gene silencing (VIGS) was performed by inoculating 30 to 35 petunia plants with each vector [27]. The treated plants were then grown under the previously described conditions.

### RNAi vector construction and petunia transformation

We inserted the intron sequence of *PhCHS* (Peaxi162Scf00536: 921496–923 395) into the pBI121 vector (https://www.ncbi.nlm.nih.gov/nuccore/AF485783.1/, accessed on January 14, 2023) at the BamHI/KpnI sites to serve as the backbone for the RNAi vector. The specific sequence of conserved regions of *PhTK1* and *PhTK2* around 500 bp was selected to amplify the antisense sequence using RNAi-PhTK1-TK2-1-F and RNAi-PhTK1-TK2-1-R primers, and the sense sequence using RNAi-PhTK1-TK2-2-F and RNAi-PhTK1-TK2-2-R primers, respectively (Table S5). The antisense fragment was constructed into the RNAi vector, and the construction was successful; then the positive-sense fragment was constructed to form a hairpin structure. This construct was transformed into *A. tumefaciens* strain GV3101 via electroporation. *Agrobacterium*-mediated transformation of petunia leaf discs was performed following the previous method [39]. After coculturing with *A. tumefaciens* cells in the coculture medium, the leaf discs were transferred to a selective medium containing kanamycin. PCR was conducted to identify putative transformants using genomic DNA extracted from the emerging shoots. Only plants exhibiting both kanamycin resistance and PCR confirmation were transferred to rooting medium for further growth. Once roots had developed, the seedlings were transplanted into soil and cultivated under the aforementioned greenhouse conditions for approximately 6 months until flowering. Transgenic petunia plants were finally validated through flower phenotype assessment and qPCR analysis.

### Total anthocyanin measurement

Petunia corollas were harvested for anthocyanin extraction and quantification following a previously described method [23]. Each treatment was assessed using three biological replicates.

### Chlorophyll content measurement

Chlorophyll was extracted from petunia leaves following a previously described method [40]. The supernatant was analyzed spectrophotometrically at 663.5 and 646.6 nm. Each treatment was assessed using three biological replicates.

### Chlorophyll fluorescence measurement

Before measurement, leaves were dark adapted for 15 min. Photosynthesis efficiency parameters were then assessed using a chlorophyll fluorescence imaging system (PlantExplorer Pro, PhenoVation, The Netherlands).

### Determination of free amino acids

The leaves from *PhTK1*-*TK2*-RNAi-30 and control plants were harvested for the determination of amino acids. The pretreatment and amino acids quantification were performed as previously described [23]. Leaf samples from *PhTK1*-*TK2*-RNAi-30 and control plants were collected, flash frozen in liquid nitrogen, and ground into a fine powder. This powder was then pretreated using a 5% solution of 5-sulfosalicylic acid dihydrate (Macklin, M327718069). After centrifugation, the supernatant was collected for amino acid analysis. The assay was conducted according to the JY/T 0576-2020 general amino acid analysis protocol using a Hitachi L-8900 amino acid analyzer equipped with a Hitachi 855-4507 column maintained at 135°C. Detection occurred at 570 and 440 nm, with elution and derivatization pump flow rates of 0.35 and 0.30 ml·min^−1^, respectively. FUJIFILM external standards (010-28164, 016-28144) were used for quantification.

### Measurement of E4P and flavonoid content

Flowers at full bloom were harvested from both *PhTK1*-*TK2*-RNAi-30 and control plants, ground into a fine powder in liquid nitrogen. The supernatant was collected after centrifugation for subsequent assay. The content of E4P and flavonoids was quantified using enzyme-linked immunosorbent assay (ELISA) kits (MEIMIAN, Suzhou, China) in accordance with the manufacturer’s instructions. The kits and catalog numbers are as follows: E4P (MM-62917O2) and flavonoids (MM-205301). Absorbance measurements were performed using a Thermo Scientific Multiskan SkyHigh microplate reader (A51119600DPC).

### Analysis of flavonoid metabolome

A total of 0.1 g of freeze-dried corollas from *PhTK1*-*TK2*-RNAi-30 and control plants was ground into a powder and incubated in 1.0 ml of 70% aqueous methanol at 4°C overnight. The petunia flower extracts were analyzed using a UPLC-ESI-MS/MS system (UPLC, SHIMADZU Nexera X2; MS, Applied Biosystems 4500 Q TRAP). Flavonoid metabolites were analyzed according to previously described methods [29].

### Statistical analysis

The statistical analysis used one-way ANOVA followed by Duncan’s multiple range test with at least three replicates. *P* values ≤0.05 were considered significant.

## Supplementary Material

Web_Material_uhaf285

## Data Availability

All data included in this study are included in this article and its supplementary information files.
