# Supplementary material for: Transketolase-mediated erythrose-4-phosphate provides an essential source for anthocyanin biosynthesis in petunia
Source: Hortic Res. 2025 Dec 22;12(12):uhaf285. doi: 10.1093/hr/uhaf285 (PMC12721374; doi:10.1093/hr/uhaf285)
Supplement: Web_Material_uhaf285 [file web_material_uhaf285.zip › Supplementary information.pdf]

Supplementary information

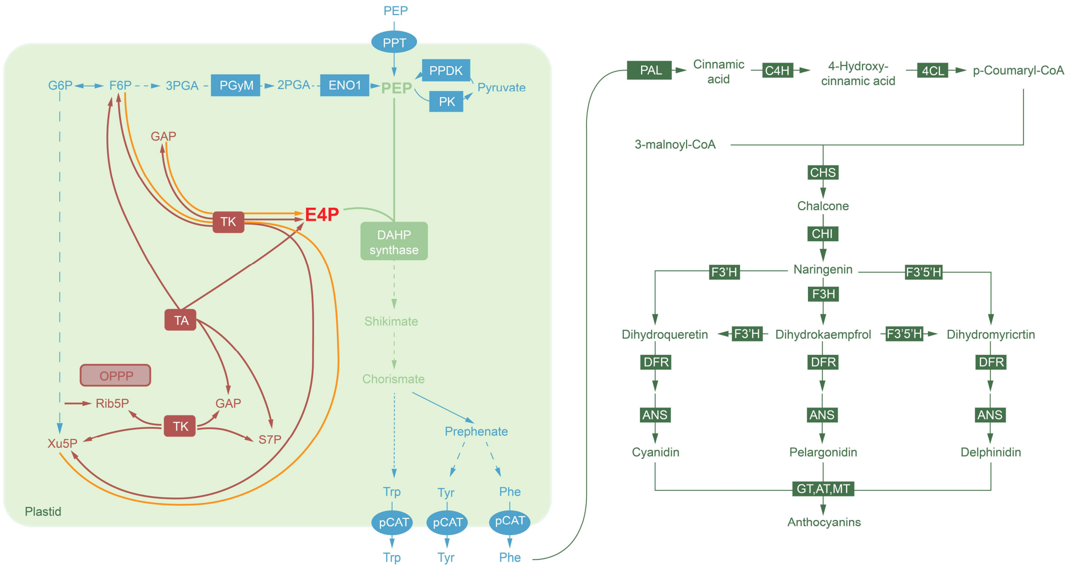

Figure S1 Diagram of transaldolase (TA) and transketolase (TK) to generate erythrose 4-phosphate (E4P) and ultimately anthocyanins, modified from Maeda & Dudareva (2012). E4P can be synthesized by TK as part of the Calvin cycle (orange lines) or by either TA or TK through the oxidative pentose phosphate pathway (OPPP, red lines). G6P, glucose 6-phosphate; F6P, fructose-6-phosphate; 3PGA, 3-phosphoglycerate; Rib5P, ribose 5-phosphate; S7P, sedoheptulose-7-phosphate; GAP, glyceraldehyde-3-phosphate; Xu5P, xylose 5-phosphate; PEP, phosphoenolpyruvate ; Phe, phenylalanine; Tyr, tyrosine; Trp, tryptophan; pCAT, plastidial cationic amino-acid transporter; DAHP synthase, 3-deoxy-d-arabino-heptulosonate-7-phosphate synthase; PAL, phenylalanine ammonia lyase; C4H, cinnamate4-hydroxy lase; 4CL, 4-coumarate CoA ligase; CHS, chalcone synthase; CHI, chalcone isomerase; F3’H, flavanone 3’-hydroxylase; F3’5’H, flavanone 3’5’-hydroxylase; F3H, flavanone3-hydroxylase; DFR, dihydroflavonol 4-reductase; ANS, anthocyanidin synthase; GT, glucosyltransferase; AT, acyltransferase;MT,methyl-transferase.

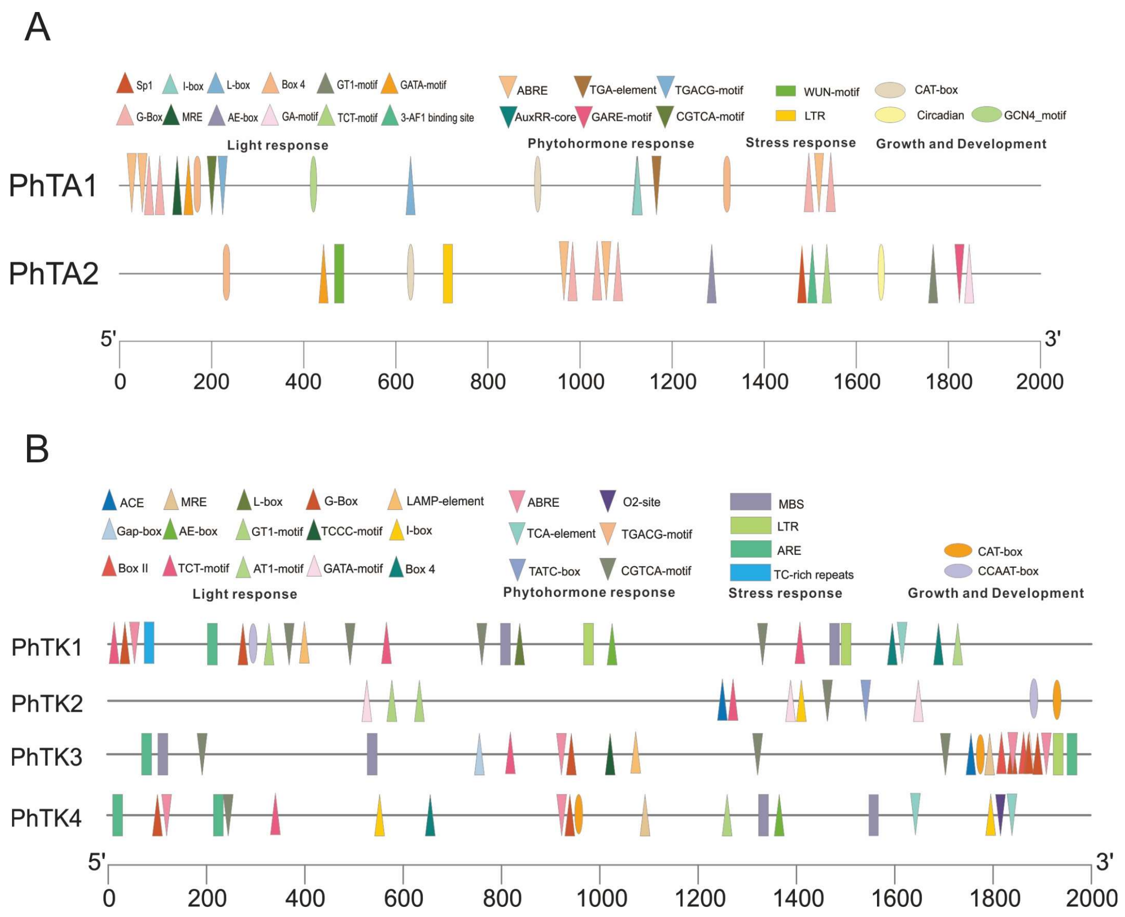

Figure S2 Promoter analysis of *PhTAs* (A) and *PhTKs* (B).

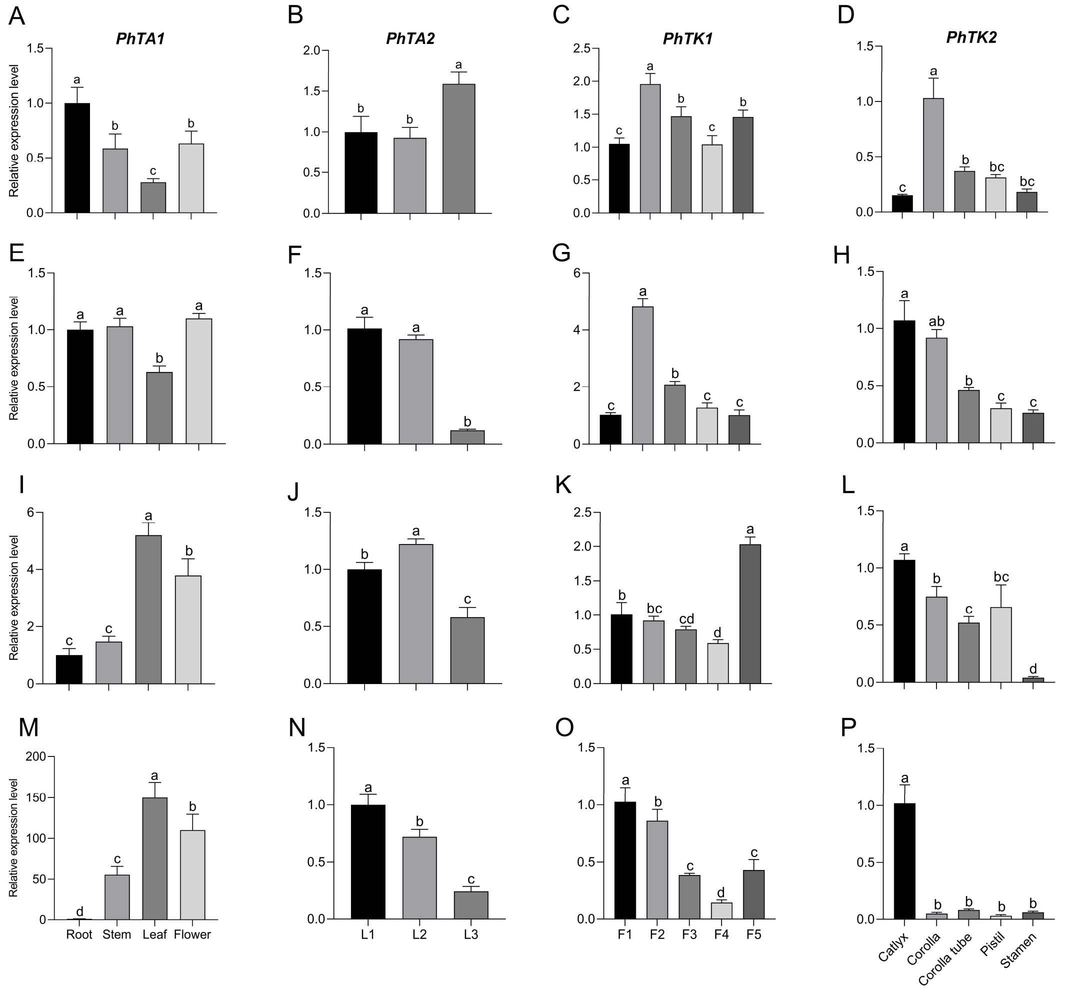

Figure S3 The expression patterns of *PhTA1*, *PhTA2, PhTK1,* and *PhTK2* were determined using quantitative real-time PCR. L1 (length 2.5 cm), L2 (4.0 cm), and L3 (7.0 cm). Flower development was divided into five stages: F1 (length 0.5 cm), F2 (1.0 cm), F3 (2.0 cm, the coloring stage of the flower), F4 (3.0 cm), and F5 (flowering stage). Data in the graphs are expressed as mean ± SD (n = 9). Different lowercase letters indicated significant differences at the P = 0.05 level.

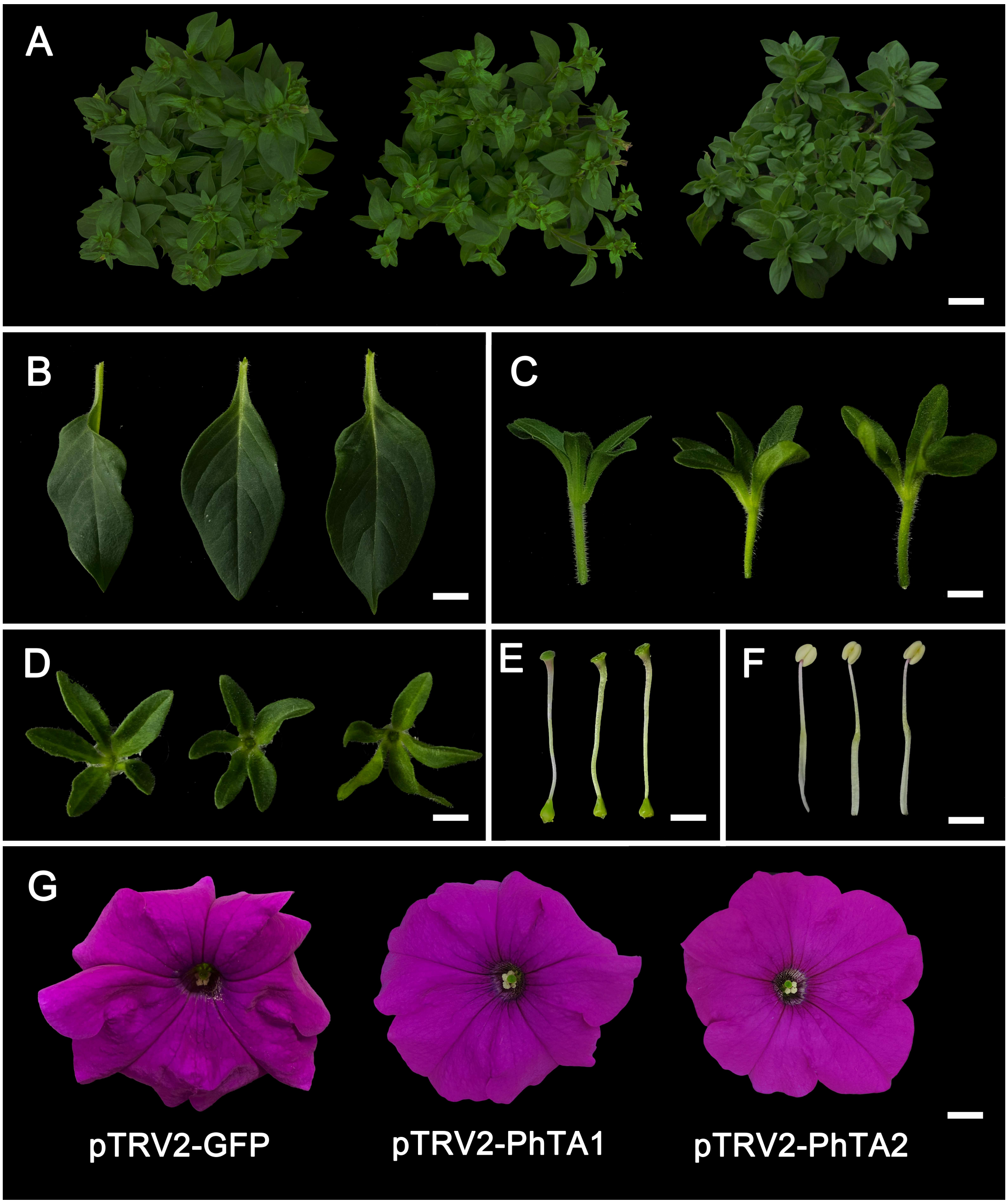

Figure S4 The phenotype of VIGS-mediated co-silencing of *PhTA1 and PhTA2.* Left to right: pTRV2-GFP (control), pTRV2-PhTA1, and pTRV2-PhTA2 plants: top view (A), leaves (B), front view of calyx (C), top view of calyx (D), pistils (E), stamens (F), and flowers (G). Scale bars=3 cm in (A), 2 cm in (B), 1 cm in (C) and (D), 0.5 cm in (E), (F), and 2 cm in (G).

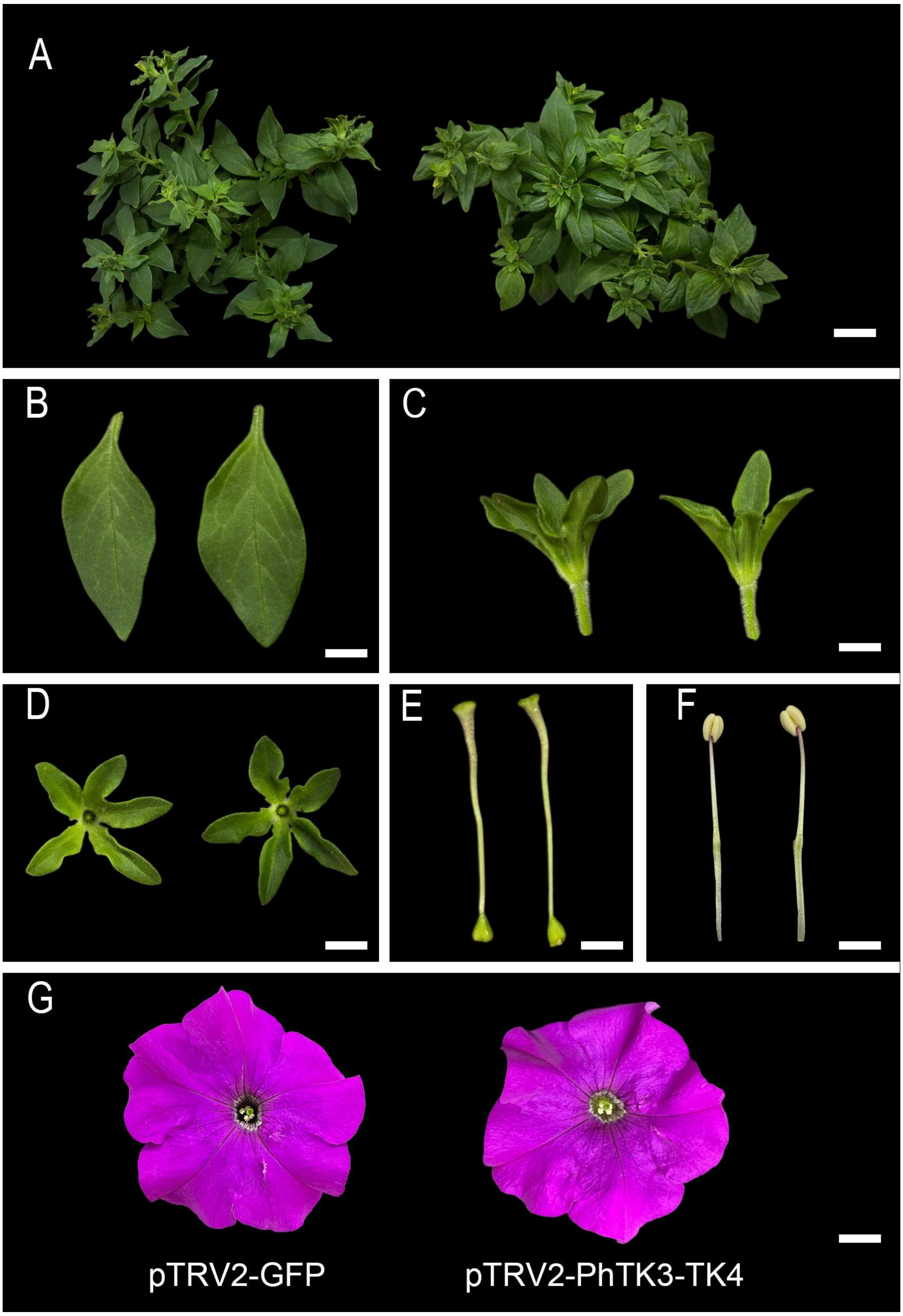

Figure S5 The phenotype of VIGS-mediated co-silencing of *PhTK3 and PhTK4.* Left to right: pTRV2-GFP (control), *PhTK3-TK4*-silenced plants: top view (A), leaves (B), front view of calyx (C), top view of calyx (D), pistils (E), stamens (F), and flowers (G). Scale bars=3 cm in (A), 2 cm in (B), 1 cm in (C) and (D), 0.5 cm in (E), (F), and 2 cm in (G).

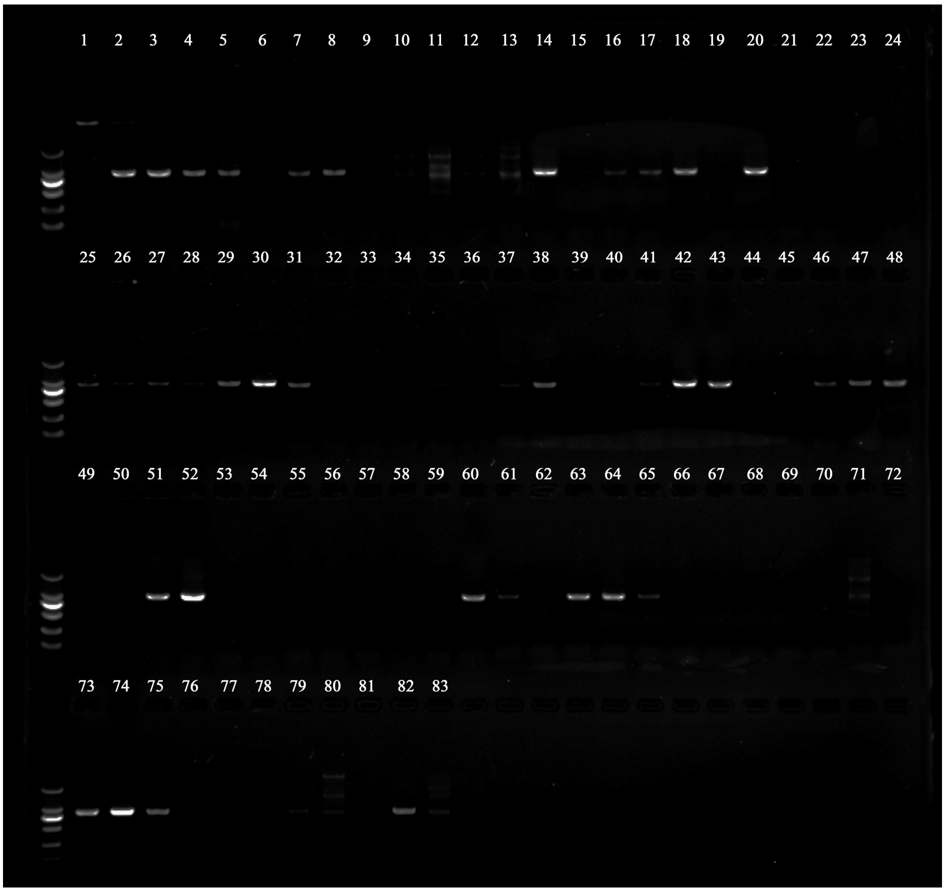

Figure S6 Electropherogram of RNAi-mediated PCR assay for *PhTK1-TK2*-RNAi-silenced plants.

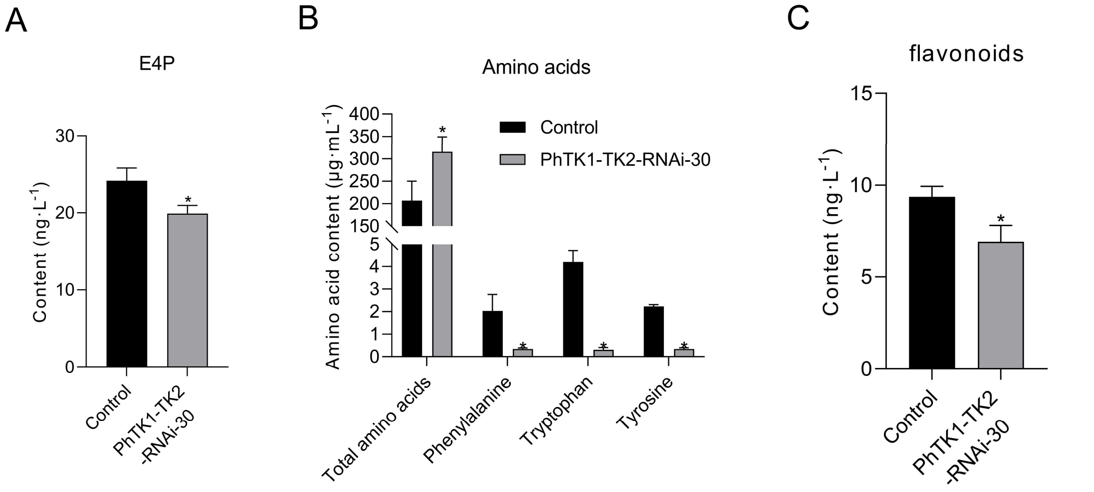

Figure S7 Contents of E4P (A), amino acids (B), and flavonoids (C) in PhTK1-TK2-RNAi-30 plants. Data are presented as mean ± SD (n = 3). Asterisk means significant difference at P = 0.05 level.

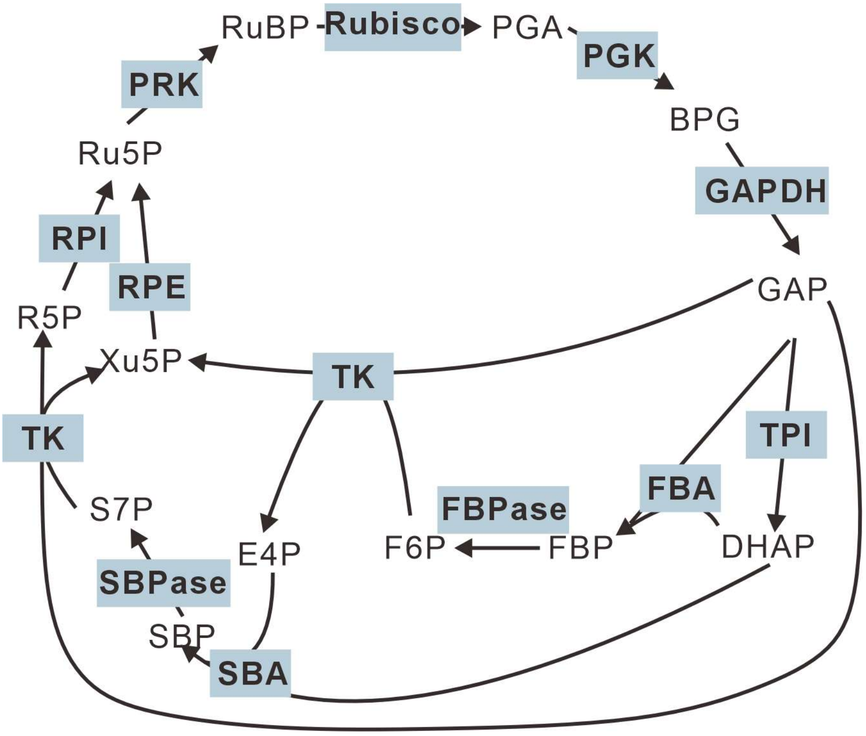

Figure S8 Diagram of the Calvin cycle modified from Ohta (2022). RuBP, ribulose-1,5-bisphosphate; PGA, 3-phosphoglycerate; BPG, 1,3-bisphosphoglycerate; GAP, glyceraldehyde-3-phosphate; DHAP, dihydroxyacetone phosphate; FBP, fructose-1,6-bisphosphate; F6P, fructose-6-phosphate; E4P, erythrose-4-phosphate; SBP, sedoheptulose-1,7-bisphosphate; S7P, sedoheptulose-7-phosphate; Xu5P, xylulose-5-phosphate; R5P, ribose-5-phosphate; Ru5P, ribulose-5-phosphate. Rubisco, ribulose-1,5-bisphosphate carboxylase/oxygenase; PGK, phosphoglycerate kinase; GAPDH, glyceraldehyde-3-phosphate dehydrogenase; TPI, triosephosphate isomerase; FBA, fructose-1,6-bisphosphate aldolase; FBPase, fructose-1,6-bisphosphatase; SBA, SBP aldolase; SBPase, sedoheptulose-1,7-bisphosphatase; TK, transketolase; RPE, ribulose-5-phosphate epimerase; RPI, ribose-5-phosphate isomerase; PRK, phosphoribulokinase.

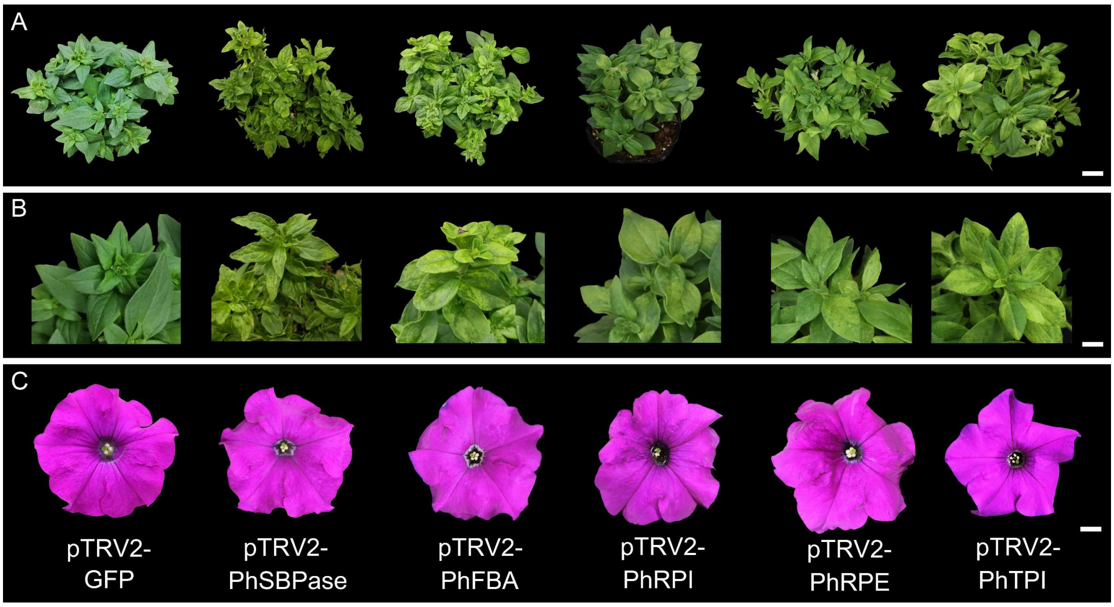

Figure S9 The phenotype of VIGS-mediated silencing of key genes in the Calvin cycle. Left to right: pTRV2-GFP (control), PhSBPase-, PhFBA-, PhRPI-, PhRPE-, and PhTPI-silenced plants: top view (A), partial enlargement (B), and flowers (C). Scale bars=5 cm in (A), 3 cm in (B), and 1 cm in (C).

Table S1 The FPKM of *PhTKs* among different stages of leaf and flower

L1, L2, and L3 represent three stages with leaf lengths of 2.5 cm, 4 cm, and 7 cm, respectively. F1, F3, and F5 represent the budding stage, coloring stage, and blooming stage of the flower, respectively.

Gene name	L1	L2	L3	F1	F3	F5
PhTK1	185.39	333.64	291.25	262.35	149.12	341.69
PhTK2	174.22	477.96	253.82	65.96	5.45	2.24
PhTK3	0.34	0.22	0.67	0.53	0.7	1.62
PhTK4	5.29	7.42	3.33	7.25	3.5	3.46

Table S2 Primers used for subcellular localization

Primer name	Primer sequences（5’ - 3’）
1403-PhTA1-F	TCACCATTTACGAACGATAGCCATGGCA ATGGCTACCATTTCTAGGCTAAC
1403-PhTA1-R	AGCTCCTCGCCCTTGCTCACCATGGACA GACTCACTAACTTGAGTGTGTTTG
1403-PhTA2-F	TCACCATTTACGAACGATAGCC ATGTCATCATCCTCATTGC
1403-PhTA2-R	AGCTCCTCGCCCTTGCTCACCATGGATA CATTTGGTGGTGGCCATATC
1403-PhTK1-F	TCACCATTTACGAACGATAGCCATGGCA ATGGCTTCTTCTTCTAGCCTCACTCTTTC
1403-PhTK1-R	AGCTCCTCGCCCTTGCTCACCATGGAAA GAAGTTCTTTAGCTGCAGCTACAACAG
1403-PhTK2-F	TCACCATTTACGAACGATAGCCATGGCT TCTTCATCTTCACTCACACTCT
1403-PhTK2-R	AGCTCCTCGCCCTTGCTCACCATGGAAG AAACTTCTTTAGCTGCAGCTACAAC

Table S3 Primers used for qPCR

Primer name	Primer sequences（5’ - 3’）
qP-CYP-F	AGGCTCATCATTCCACCGTGT
qP-CYP-R	TCATCTGCGAACTTAGCACCG
qP-PhTA1-F	TGCAGGAGAAGGCAAACACAC
qP-PhTA1-R	CATTACAACGCAGCAGCACAC
qP-PhTA2-F	CCAACATGCAATACCAGGATGTTC
qP-PhTA2-R	CCTATTGCGTGGAAGACTCACC
qP-PhTK1-F	TGACAGATTCGGTGCTAGTGC
qP-PhTK1-R	TTAGCTGCAGCTACAACAGCC
qP-PhTK2-F	TGGCTCTTCTTCATCTCCTTCAC
qP-PhTK2-R	CGAATGGAGTTATGTTTAACGCCG

Table S4 Primers used for VIGS

Primer name	Primer sequences（5' - 3'）
pTRV2-PhTA1-F	GTGAGCTCGGTACCGGATCCGTCCTAGATGG GAGGCTTTG
pTRV2-PhTA1-R	TGAGTAAGGTTACCGAATTCGTGTGTTTGCC TTCTCCTG
pTRV2-PhTA2-F	GTGAGCTCGGTACCCAAACAATTCCTCAAC CCGCTT
pTRV2-PhTA2-R	TGAGTAAGGTTACCGAATTCCAGTGGTGATC CTGAAGTTGAAAG
pTRV2-PhTK1-TK2-F	GTGAGCTCGGTACCGGATCCTTGAAGGAAC AATGAAGGGTGG
pTRV2-PhTK1-TK2-R	TGAGTAAGGTTACCGAATTCCCTCTGCGGTA ATTCCAAACTC
pTRV2-PhTK1-F	ACGCGTGAGCTCGGTACCGGATCCAGTTTG AGCAGCGGAGC
pTRV2-PhTK1-R	TCTGTGAGTAAGGTTACCGAATTCGTGCATG GGAAGCAATCATG
pTRV2-PhTK2-F	ACGCGTGAGCTCGGTACCGGATCCTTGAGC AGTAAAGCCACCAG
pTRV2-PhTK2-R	TCTGTGAGTAAGGTTACCGAATTCGAGCATG TCATCAAACTAGCC
pTRV2-PhTK3-TK4-F	CGTGAGCTCGGTACCGGATCCTGGCACTTG GAATGGCTG
pTRV2-PhTK3-TK4-R	GTGAGTAAGGTTACCGAATTCCCTCCATAGA ACAGCCATCAC

Table S5 Primers used for RNAi

Primer name	Primer sequences（5' - 3'）
RNAi-PhTK1-TK2-1-F	AGAACACGGGGGACTCTAGAGCACCGA ATCTGTCAATTCC
RNAi-PhTK1-TK2-1-R	TTACACATGCGCTGGGATCCTCGTGAAC ATGGTATGGGAG
RNAi-PhTK1-TK2-2-F	GCAGGTGACAAATCGGTACCTCGTGAAC ATGGTATGGGAG
RNAi-PhTK1-TK2-2-R	TGGGTACATCTCGAGGGTACCGCACCGA ATCTGTCAATTCC

References

Maeda H, Dudareva N (2012) The Shikimate pathway and aromatic amino acid biosynthesis in Plants. Annu Rev Plant Biol **63**: 73–105

Ohta J (2022) A novel variant of the Calvin–Benson cycle bypassing fructose bisphosphate. Sci Rep. doi: 10.1038/s41598-022-07836-7
